# TLR4 antagonist FP7 inhibits LPS-induced cytokine production and glycolytic reprogramming in dendritic cells, and protects mice from lethal influenza infection

**DOI:** 10.1038/srep40791

**Published:** 2017-01-20

**Authors:** Laure Perrin-Cocon, Anne Aublin-Gex, Stefania E. Sestito, Kari Ann Shirey, Mira C. Patel, Patrice André, Jorge C. Blanco, Stefanie N. Vogel, Francesco Peri, Vincent Lotteau

**Affiliations:** 1 CIRI, International Center for Infectiology Research, Université de Lyon, Lyon, France; 2 Inserm, U1111, Lyon, France; 3 Ecole Normale Supérieure de Lyon, Lyon, France; 4 Université Lyon 1, Centre International de Recherche en Infectiologie, Lyon, France; 5 CNRS, UMR5308, Lyon, France; 6 Department of Biotechnology and Biosciences, University of Milano-Bicocca, Piazza della Scienza, 2, 20126 Milano, Italy; 7 Department of Microbiology and Immunology, University of Maryland, School of Medicine, Baltimore, Maryland 21201, USA; 8 Sigmovir Biosystems Inc., Rockville, MD, USA

## Abstract

Dysregulated Toll-like receptor (TLR)-4 activation is involved in acute systemic sepsis, chronic inflammatory diseases, such as atherosclerosis and diabetes, and in viral infections, such as influenza infection. Thus, therapeutic control of the TLR4 signalling pathway is of major interest. Here we tested the activity of the small-molecule synthetic TLR4 antagonist, FP7, *in vitro* on human monocytes and monocyte-derived dendritic cells (DCs) and *in vivo* during influenza virus infection of mice. Our results indicate that FP7 antagonized the secretion of proinflammatory cytokines (IL-6, IL-8, and MIP-1β) by monocytes and DCs (IC_50_ < 1 μM) and prevented DC maturation upon TLR4 activation by ultrapure lipopolysaccharide (LPS). FP7 selectively blocked TLR4 stimulation, but not TLR1/2, TLR2/6, or TLR3 activation. TLR4 stimulation of human DCs resulted in increased glycolytic activity that was also antagonized by FP7. FP7 protected mice from influenza virus-induced lethality and reduced both proinflammatory cytokine gene expression in the lungs and acute lung injury (ALI). Therefore, FP7 can antagonize TLR4 activation *in vitro* and protect mice from severe influenza infection, most likely by reducing TLR4-dependent cytokine storm mediated by damage-associated molecular patterns (DAMPs) like HMGB1.

Among the first receptors activated during host-pathogen interactions are members of the Toll-like Receptor (TLR) family, which can detect microbial products and induce innate and adaptive immune responses^1^. TLRs are pattern recognition receptors (PRR) that recognize pathogen-associated molecular patterns (PAMPs). Among them, TLR4 is the sensor of the Gram-negative bacteria endotoxin, lipopolysaccharide (LPS). TLR4 is mainly expressed on monocytes, dendritic cells (DCs), and macrophages (MΦs). LPS sensing and TLR4 activation are sequential processes, initiated by transfer of LPS monomers from aggregates in solution to LPS-binding protein (LBP), and subsequently to cluster of differentiation 14 (CD14, GPI-linked or soluble), and finally, to myeloid differentiation factor 2 (MD-2) that associates with TLR4 noncovalently^2^,^3^. Appropriate ligand binding to MD-2 results in dimerization of two TLR4.MD-2 complexes, thus forming the activated heterodimer (LPS.MD-2.TLR4)_2_ on the plasma membrane^4^. The involvement of the two co-receptors, CD14 and MD-2, in the process of ligand binding to MD-2 leading to receptor activation is unique to TLR4 and ensures a high degree sensitivity to endotoxin (detected at pM concentration). TLR4 is also the only TLR that activates both the MyD88-dependent pathway resulting in NF-κB and AP-1 activation and the MyD88-independent pathway involving the adaptors TRAM and TRIF, triggering IRF3 activation. The co-receptor CD14 has also been shown to be essential for initiating endocytosis of (TLR4.MD-2.LPS)_2_ heterodimers and the subsequent activation of TRAM-TRIF pathway leading to the production of type I IFN^5^. Many other microbial ligands of TLR4 have been described such as the Mouse Mammary Tumor Virus envelope protein, respiratory syncytial virus (RSV) Fusion (F) protein, the vesicular stomatitis virus G glycoprotein, and Ebola virus GP^6^,^7^,^8^,^9^. TLR4 can be also activated by damage-associated molecular patterns (DAMPs) derived from damaged, necrotic, or infected tissues. While different LPS types with inflammatory activities share a conserved lipid A moiety with chemical determinants that ensure optimal interaction with CD14 and MD-2 (5 or 6 lipophilic fatty acid chains attached to a disaccharide backbone, and one or two phosphate groups), DAMPs are chemically diverse and range from proteins (*e.g.*, high-mobility group box 1, HMGB1) to oxidized phospholipids (oxPL)^10^. The molecular mechanism of TLR4 activation by DAMPs and the role of CD14 and MD-2 in the sensing of these molecules are not entirely understood; however, a more general role for CD14 and MD-2 as PAMP and DAMP co-receptors has begun to emerge^11^. DAMPs have been implicated in a large array of pathologies caused by TLR4 activation including atherosclerosis^12^, rheumatoid arthritis (RA)^13^, neuroinflammation, neuropathic pain^14^, and neurodegenerative diseases such as Alzheimer’s disease (AD), amyotrophic lateral sclerosis (ALS)^15^, ischemia/reperfusion, trauma^16^, and haemorrhage^17^.

While TLR4 is the specific signalling receptor for bacterial LPS, recent data suggest a central role for TLR4 signalling in the pathology associated with viral infections. TLR4 is involved in RSV infection^18^, hepatitis C^19^, viral sepsis, tissue oxidative damage, and pulmonary syndromes caused by influenza^20^. Massive cytokine production (“cytokine storm”) is often associated with sepsis and septic shock following overwhelming TLR4 stimulation by Gram negative bacteria^21^,^22^,^23^. There are striking similarities in the syndromes caused by bacterial sepsis and by Ebola virus (EBOV) characterized by systemic inflammation, endothelial dysfunction, coagulopathy, vascular leak, shock, and organ failure^24^,^25^. Interestingly, EBOV soluble glycoproteins were found to activate human DCs and MΦs through a TLR4-dependent process^24^.

Metabolic reprogramming has emerged as a key checkpoint for the activation of innate immune cells such as murine MΦs and DCs^26^, for which TLR4 activation results in the preferential use of glycolysis, rather than mitochondrial catabolic pathways^27^,^28^,^29^,^30^. This process recalls the Warburg effect observed in tumour cells^31^. Pathogens may exploit TLR4-dependent regulation of glycolysis to respond to increased energetic demands or to modulate the function of antigen-presenting cells. Although a Warburg-like effect has been reported in virus-infected cells^32^,^33^,^34^,^35^, it is not yet known whether this relates to alterations of the TLR4 signalling pathway by PAMPs or host-derived DAMPs.

Eritoran (also known as E5564, Fig. 1b) is a potent and selective synthetic TLR4 antagonist developed by Eisai Inc. that mimics the chemical structure of lipid A (Fig. 1a)^36^. Despite the potent anti-sepsis activity of Eritoran in animal models, and despite its excellent safety profile in humans, it failed in a Phase III randomized controlled trial for all cause sepsis^37^.

Multiple lung insults by chemical (*e.g*., acid) and diverse microbial agents (*e.g*., influenza viruses, SARS) cause high lethality due to acute lung injury (ALI)^20^. Imai *et al*. suggested that such insults trigger reactive oxygen species generating host-derived oxidized phospholipid 1-palmitoyl-2-arachidonyl-phosphatidylcholine (OxPAPC) that, in turn, stimulates a common TLR4-, TRIF-, and IL-6-dependent pathway in MΦs leading to ALI^20^. Treatment of influenza virus-infected mice with Eritoran blocked influenza virus-induced lethality and ALI^38^. The mechanism of action of Eritoran is suspected to be based on the inhibition of TLR4 activation by endogenous DAMPs produced in the late phase of viral infection^38^,^39^.

Although treatment of bacterial sepsis with TLR4 antagonists may be limited to selected patients, there is a large potential of development for TLR4-oriented therapeutics to target sterile or microbially-induced inflammation. In an effort to characterize a synthetic small-molecule drug candidate that can modulate innate immunity, we developed the potent TLR4 antagonist FP7^40^ with the chemical structure of a diacylated, di-phosphorylated monosaccharide (Fig. 1c). We present here the activity of this molecule *in vitro* and in influenza-virus infected mice.

## Results

### Inhibition of LPS-induced activation of human monocytes and maturation of DCs by FP7

**Figure 2:**
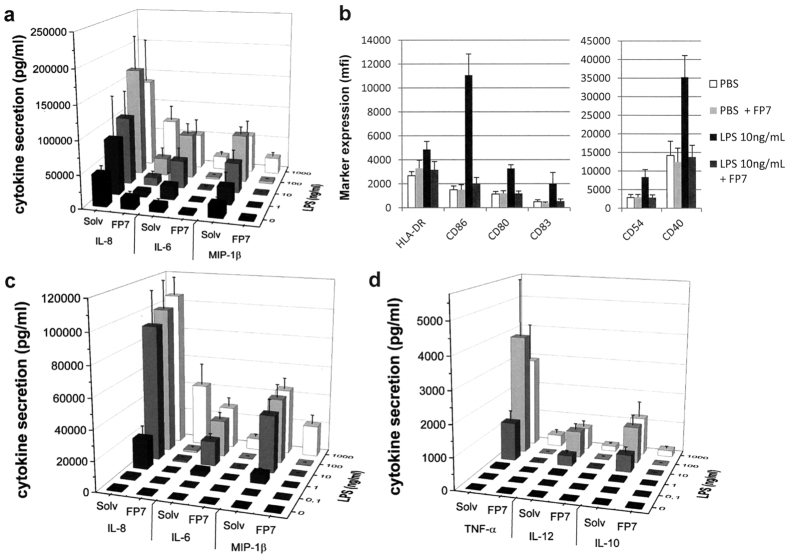
FP7 antagonizes LPS-induced human monocyte stimulation and DC maturation. (**a**) Monocytes were isolated from peripheral human blood, stimulated for 24 h with increasing amounts of LPS or the same volume of PBS as control, in the presence of 10 μM FP7 or solvent (Solv). Data represents mean cytokine secretion in monocyte supernatants from 4 independent experiments. (**b**–**d**) Dendritic cells (DCs) were differentiated from human peripheral blood monocytes for 6 days. DCs were stimulated for 24 h with increasing amounts of LPS or the same volume of PBS as control, in the presence of 10 μM FP7 or solvent. Surface expression of DC maturation markers was monitored by flow cytometry and means ± SEM of fluorescence intensity are shown (**b**). Cytokine secretion was assayed in DC supernatants by Cytometric Bead Array (CBA). Data represents mean cytokine secretion from 6 independent experiments.

Monocytes that circulate in the peripheral blood play an important role in the detection of pathogens and danger signals by secreting cytokines and chemokines and are precursors of tissue-infiltrating DCs that are potent antigen-presenting cells during infection^41^,^42^,^43^. *In vitro*, DCs can be differentiated from blood monocytes by incubation with GM-CSF and IL-4 for 6 days^44^. Both monocytes and monocyte-derived DCs express TLR4 and stimulation of TLR4 by LPS induces the secretion of proinflammatory cytokines, although the nature of the cytokine response varies according to the cell type (Fig. 2). TLR4 stimulation of DCs induces their phenotypic and functional maturation^19^. The inhibitory action of FP7 was thus tested on these primary cells. Cells were pre-treated with FP7 15 minutes prior to LPS stimulation for 24 h. FP7 almost completely blocked induction of IL-8, IL-6, and MIP-1β, 3 of the major cytokines secreted by monocytes stimulated with LPS (Fig. 2a and Supplementary Fig. S1a). DCs stimulated by LPS showed a classic phenotype of human mature DC (mDC) with a strong induction of CD86 and CD40 expression and increased expression of HLA-DR, CD80, CD83 and CD54 (Fig. 2b). LPS-stimulated DCs secrete high levels of IL-8, IL-6, MIP-1β and TNFα, as well as IL-12 and IL-10 that are important indicators of their function (Supplementary Fig. S1b). FP7 abrogated the enhanced expression of all these phenotypic markers triggered by LPS. After DC differentiation, FP7 treatment also prevented LPS-induced secretion of IL-8, IL-6, MIP-1β, TNF-α, IL-12 p40, and IL-10 up to 100 ng/ml of LPS and inhibited cytokine secretion induced by 1000 ng/ml (Fig. 2c,d). Thus, FP7 prevents LPS-induced maturation of DCs.

### Dose-dependent inhibition of TLR4 signalling by FP7

**Figure 3:**
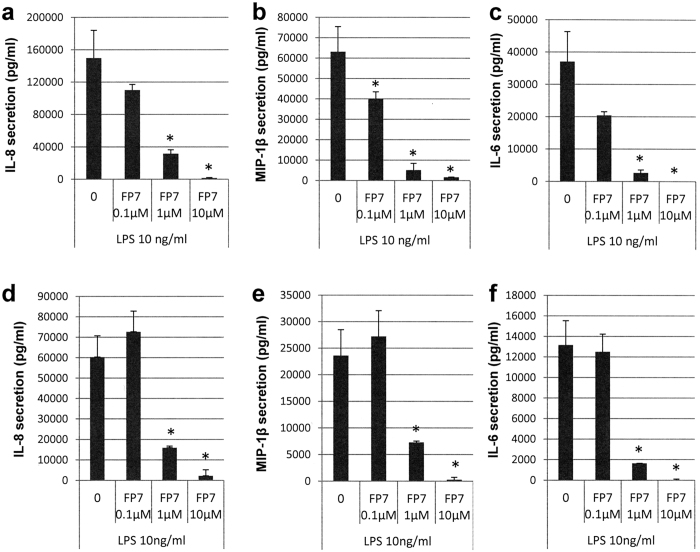
Dose-dependent inhibition of monocyte and DC activation by FP7. Human monocytes (**a**–**c**) or DCs (**d**–**f**) were stimulated for 24 h with 10 ng/ml LPS, in the absence or presence of increasing amounts FP7. IL-8, MIP-1β, and IL-6 were assayed in supernatants of a representative experiment. *P < 0.05 compared to LPS-stimulated cells.

Previous results have shown that FP7 antagonized LPS signalling in HEK-Blue™-hTLR4 cells stably co-expressing TLR4, MD-2, and CD14 with an IC_50_ of 0.46 μM for 10 ng/ml of LPS^40^. To evaluate the IC_50_ of FP7 on primary cells, monocytes and DCs were pre-treated with increasing doses of FP7 for 15 minutes before stimulation with 10 ng/ml of LPS. IL-6, IL-8, and MIP-1β secretion was then assayed in supernatants collected 24 h post-stimulation. The results indicate that FP7 inhibits the secretion of proinflammatory cytokines both in monocytes and DCs in a dose-dependent manner (Fig. 3). For monocytes, FP7 IC_50_ was 0.06 μM for IL-6, 0.13 μM for IL-8, and 0.07 μM for MIP-1β secretion. For DCs, FP7 IC_50_ was 0.22 μM for IL-6, 0.44 μM for IL-8, and 0.32 μM for MIP-1β secretion. These results correlate with the previous data obtained with HEK-Blue™-hTLR4 cells^40^. Monocyte and DC viability measurements showed that FP7 was not toxic at the maximal concentration of 10 μM (Supplementary Fig. S2). This concentration of FP7 was thus used in further experiments.

### FP7 selectively antagonizes TLR4 and has no effect on TLR1/2, TLR2/6, and TLR3 signalling

**Figure 4:**
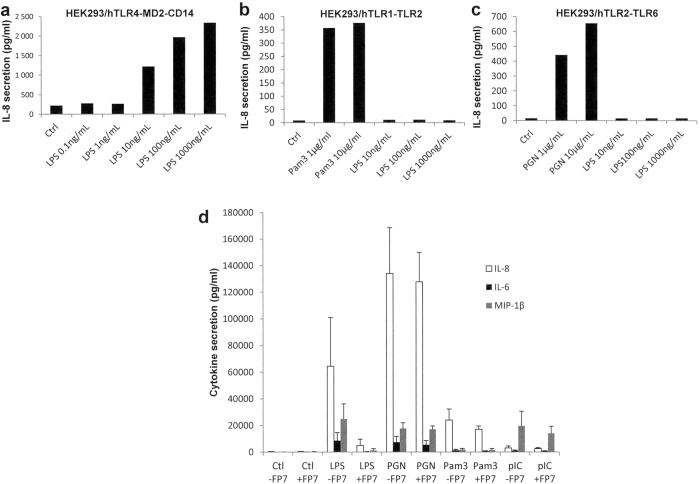
FP7 selectively antagonizes TLR4. (**a**–**c**) HEK293/hTLR4-MD2-CD14, HEK293/hTLR1-TLR2, HEK293/hTLR2-TLR6 cells were treated 24 h with ultrapure LPS, peptidoglycan (PGN), or Pam_3_CSK_4_ (Pam3). Supernatants were collected and IL-8 secreted in response to TLR stimulation was assayed. (**d**) DCs were stimulated for 24 h with 10 ng/ml ultrapure LPS or 10 μg/ml PGN, PAM3, or pIC in the absence or presence of 10 μM FP7. Control cells (Ctl) received PBS instead of TLR ligand. Cytokine secretion was assayed in DC supernatants means ± SEM from 3 independent experiments are shown.

TLR4 and TLR2 receptors share the capacity to be activated by lipid ligands. The selectivity of FP7 for TLR4 had not been investigated previously^40^. TLR2 forms a heterodimer with TLR1 to bind triacylated lipopeptides^45^ and with TLR6 to recognize diacylated lipopeptides^46^. HEK293 cells co-expressing human TLR4 with MD2 and CD14 (HEK293/hTLR4-MD2-CD14), or TLR1 and TLR2 (HEK293/hTLR1-TLR2), or TLR2 and TLR6 (HEK293/hTLR2-TLR6) were used to verify that ultrapure LPS could activate TLR4 only and not TLR1/2- or TLR2/6-expressing cells (Fig. 4a–c). Endotoxin-free TLR1/2 ligand Pam_3_CSK_4_ (Pam3), TLR2/6 ligand peptidoglycan (PGN), and TLR3 ligand double-stranded RNA poly(I:C) (pIC) failed to stimulate TLR4-expressing cells (data not shown). The effect of DC pre-treatment with 10 μM FP7 prior to activation by LPS, Pam3, PGN, and pIC was analysed. Secretion of IL-6, IL-8, and MIP-1β was measured in the supernatants at 24 h post-stimulation. FP7 strongly antagonized the stimulation of DCs by TLR4 ligand, whereas it did not affect the stimulation by TLR1/2, TLR2/6, or TLR3 ligands (Fig. 4d).

### FP7 antagonizes TLR4-induced metabolic reprogramming in DCs

**Figure 5:**
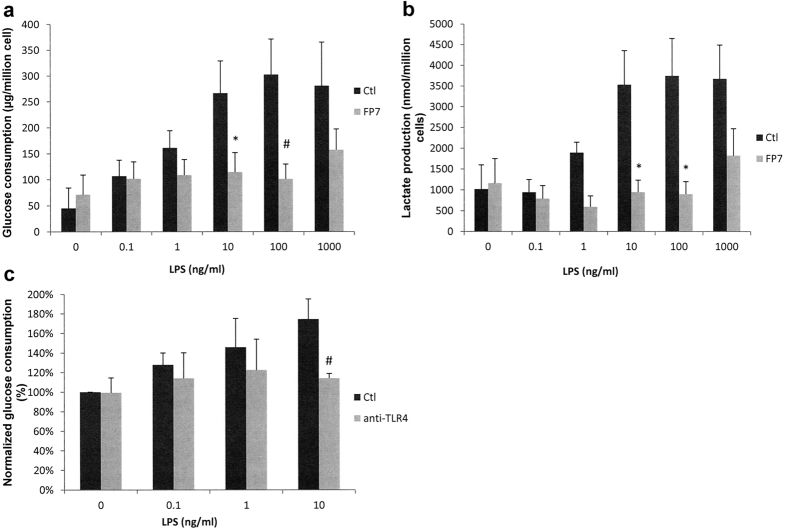
TLR4 stimulation increases glucose consumption and lactate production by human DCs. DCs were seeded at 1 × 10^6^ cells/ml in fresh medium and stimulated for 24 h with increasing amounts of LPS, in the absence or presence of 10 μM FP7 (**a**,**b**). Control cells (Ctl) received solvent instead of FP7. Glucose consumption and lactate production were monitored using enzymatic detection kits. Means ± SEM from 5 or 6 experiments are shown. (**c**) DCs were incubated with 10 μg/ml TLR4-blocking antibody or isotype control antibody 1 h prior to LPS addition. Glucose consumption was normalized to non-stimulated control DCs and means ± SEM from 3 experiments are shown. *P < 0.05; ^#^P < 0.01 FP7 compared to Ctl cells.

Previous studies found that LPS stimulation of murine bone marrow-derived DCs induced a glycolytic burst required for DC maturation^27^,^28^,^29^. We investigated whether this metabolic regulation could be observed in monocyte-derived human DCs. Differentiated DCs were seeded in fresh medium prior to LPS stimulation for 24 h. Glucose consumption and lactate production were monitored to assess the input and output rates of glycolysis. We found that LPS stimulation increased both the glucose consumption and the lactate production by DCs, indicative of an enhanced glycolytic flux (Fig. 5a,b). Treatment of cells with FP7 prior to LPS stimulation inhibited these metabolic changes induced by LPS up to 100 ng/ml. At the highest LPS concentration, FP7 could not completely block the increase of the glycolytic flux, consistent with cytokine secretion data (Fig. 2). TLR4-specific blocking antibody also prevented the increase of glucose consumption induced by LPS (Fig. 5c). These results indicate that TLR4 stimulation induced an increased glycolytic flux in monocyte-derived human DCs that can be inhibited by FP7.

### Metabolic reprogramming is necessary for DC maturation

**Figure 6:**
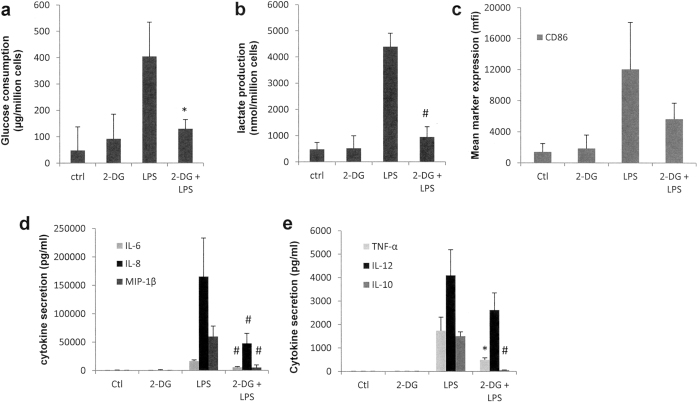
Glycolysis inhibition restrains LPS-induced DC maturation. DCs were seeded at 1 × 10^6^ cells/ml in fresh medium and stimulated for 24 h with 10 ng/ml LPS, in the absence or presence of 2.5 mM 2-deoxy-glucose (2-DG). Control cells (Ctl) received PBS only. Glucose consumption and lactate production were monitored using enzymatic detection kits (**a,b**). Surface expression of DC maturation marker CD86 was monitored by cell flow cytometry (**c**) and cytokine secretion was assayed in DC supernatants (**d**,**e**). Means ± SEM from 3 experiments are shown. *P < 0.05; ^#^P < 0.01 compared to LPS-stimulated cells.

The first rate-limiting enzyme of glycolysis is the hexokinase, and 2-deoxy-glucose (2-DG) is an allosteric inhibitor of this enzyme that is commonly used to inhibit glucose metabolism. Treatment of DC with a non-toxic low dose of 2-DG strongly inhibited LPS-induced glucose consumption and lactate production (Fig. 6a,b), as expected. By preventing the glycolytic burst induced by LPS, 2-DG also altered DC maturation as indicated by the reduced expression of the costimulatory molecule CD86 (Fig. 6c) and by the reduced secretion of cytokines triggered by LPS (Fig. 6d,e). Therefore, metabolic reprogramming of DC upon TLR4 stimulation is required for a complete maturation process of DCs. In monocytes, glucose consumption was slightly increased upon LPS stimulation and this could be inhibited by the TLR4 antagonist FP7 (Supplementary Fig. S3). This modest regulation by TLR4 seemed to be correlated with the high level of glucose consumption of these cells without stimulation.

### FP7 blocks influenza-induced lethality

**Figure 7:**
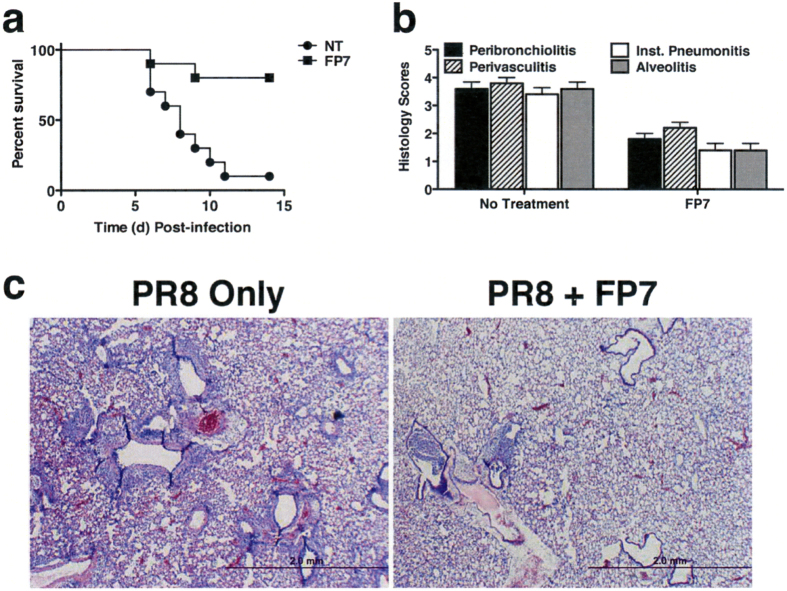
FP7 treatment protects mice from lethal influenza challenge. C57BL/6 J mice were infected with mouse-adapted influenza, strain PR8 (~7500 TCID_50_, i.n.; ~LD90). Two days later, mice received vehicle (saline; i.v.), or FP7 (200 μg/mouse; i.v.) once daily from days 2 to 6 post-infection. (**a**) Mice were monitored daily for survival for 14 days (5 mice/treatment group) (**a**). (**b**) On day 7 post-infection, another group of 5 mice per treatment were euthanized and lungs were extracted and stained for histopathology and examined for tissue damage, necrosis, apoptosis, and inflammatory cellular infiltration. Mean histopathology scores for each group were determined blindly as previously described^38^ (n = 5 mice/treatment group). (**c**) Representative H&E-stained lung sections are shown.

Previous studies reported that influenza-induced lethality due to ALI was secondary to the cytokine storm triggered by the stimulation of TLR4 by host-derived DAMPs including OxPAPC and HMGB1^20^,^39^. Interestingly, the TLR4 antagonist Eritoran protected mice from lethal influenza infection and blunted ALI^38^,^39^. We therefore tested the activity of FP7 during a lethal influenza challenge. WT mice were infected with mouse-adapted influenza strain PR8 and treated with vehicle (saline; NT), or FP7 for 5 consecutive days starting on day 2 post-influenza challenge (days 2–6). Treatment of PR8-infected mice with FP7 resulted in significant protection (P < 0.01; Fig. 7a). Haematoxylin and eosin-stained lung tissue sections were analysed and scored blindly for peribronchiolitis, interstitial pneumonia, perivasculitis, and alveolitis. FP7 treatment prevented lung pathology induced by influenza infection (Fig. 7b,c).

**Figure 8:**
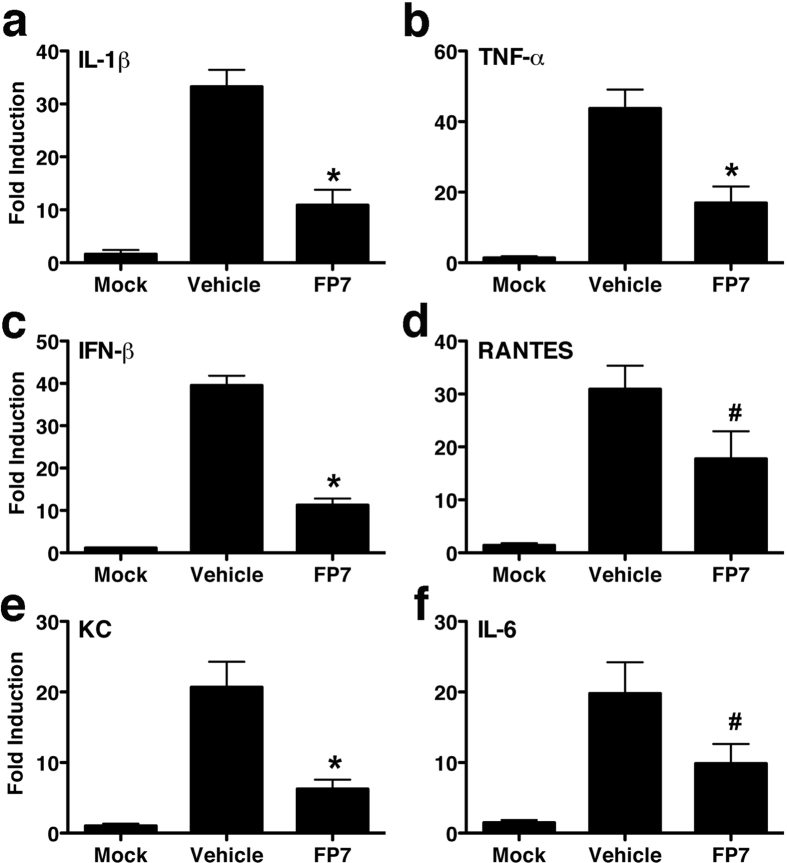
FP7 treatment suppresses influenza-induced cytokine gene expression. Mice were treated as in Fig. 7 and euthanized on day 7 post-infection (5 mice/treatment group). Lungs were processed for total RNA and subjected to qRT-PCR for detection of specific gene expression. * P < 0.01; ^#^P < 0.05.

Influenza induces expression of cytokines, *i.e.*, TNFα, IL-1β and IFN-β and chemokines such as KC (murine IL-8) that is inhibited by Eritoran therapy^38^,^39^. To assess the effect of FP7 on PR8-induced cytokine gene expression, mice were infected at day 0 and treated daily for 5 days with FP7 starting 2 days post-infection and were sacrificed on day 7 post-infection. Total RNA was extracted from lung homogenates and gene expression measured by qRT-PCR. FP7 led to a significant reduction in influenza-induced gene expression for TNF-α, IL-1β, IFN-β, KC (P < 0.01; Fig. 8a–c), IL-6, and RANTES (P < 0.05; Fig. 8d). These data were confirmed and extended by measuring cytokine protein levels and HMGB1 levels in lung homogenates from mice that were PR8-infected and treated with vehicle only or with FP7. Table 1 shows that protein levels of IL-1β, TNF-α, IFN-β, IL-6, and KC were also greatly inhibited in lungs of mice infected and treated with FP7. In these same lung homogenates, HMGB1 levels were significantly decreased in PR8-infected, FP7-treated mice (Table 1). Finally, PR8 titers in these same lung homogenates were found to be significantly less in the FP7-treated mice at day 7 post-infection (log (Vehicle-treated) titer = 5.27 ± 0.15 *vs*. log (FP7-treated) titer = 4.1 ± 0.39; p = 0.0225, 2-tailed Student’s *t* test).

## Discussion

Results obtained in this study indicate that FP7 can antagonize TLR4 activation *in vitro* and *in vivo*. FP7 antagonizes both PAMP and DAMP triggering of TLR4, inhibiting cytokine production by monocytes and DCs *in vitro* and influenza-induced cytokine gene expression *in vivo*^38^,^39^. We have shown that FP7 inhibits TLR4 signalling in a concentration-dependent manner in both monocytes and DCs, preventing the induction of cytokine secretion in both cell types and the phenotypic maturation of DCs. The IC_50_ of cytokine production inhibition by FP7 was estimated below 1 μM in both monocytes and DCs. No significant inhibition of TLR1/2 or TLR2/6 activation by their selective ligands was detected, indicating that FP7 is a selective antagonist of TLR4 even at the highest dose of 10 μM. FP7 is a lipid A analogue, shown in previous studies to bind to MD-2 and CD14 and inhibit LPS-induced TLR4 signalling^40^. Although its structure might be expected to interact with TLR2/6, that detects diacylated disaccharides, it is clear from our data that the inhibition is TLR4-specific, suggesting a more precise molecular interaction with MD-2.

The TLR4 antagonistic activity of FP7 has been characterized on human monocytes and DCs that are essential cells for the regulation of innate immunity and inflammation. These innate immune cells reside in different tissues and fulfill various functions. Monocytes recruited into inflamed tissues can differentiate to DCs or MΦs depending on the local micro-environment. DCs can be stimulated to mature DCs by exogenous pathogen-derived molecules or endogenous danger signals released by cells that are stressed, infected, or necrotic^47^,^48^,^49^. During the maturation process, DCs acquire enhanced abilities to present antigens resulting from enhanced capturing activity, increased expression of major histocompatibility complex (MHC) antigens, and induced expression of genes encoding co-stimulation molecules and cytokines. Mature DCs can migrate to lymph nodes where they present antigens and prime naïve T cells^43^, whereas MΦs are resident cells that have high phagocytic capacities, secrete cytokines, and present antigens to tissue-infiltrating T cells. Our results indicate that FP7 inhibits TLR4-mediated monocyte and DC activation and this could prevent excessive TLR4 signalling involved in inflammatory pathologies.

The importance of metabolic regulation in the control of DCs and MΦs functions has recently emerged from several studies^50^. We found in this study that TLR4 stimulation of human monocyte-derived DCs increased both glucose consumption and lactate production that are indicators of an increased glycolytic activity. Preventing the glycolytic burst using an hexokinase inhibitor strongly reduced the TLR4-dependent induction of the costimulatory molecule CD86 and the secretion of cytokines, indicating that upregulation of glycolysis is required for proper DC maturation. These results are in agreement with previous studies showing that TLR4 signalling induced in murine bone marrow-derived DCs a transition of cellular metabolism to aerobic glycolysis, similar to the Warburg effect described in cancer cells^27^,^28^,^29^. A similar metabolic switch was also described for murine MΦs stimulated by LPS and resulting in a proinflammatory “M1” phenotype, again consistent with the effect of 2DG on MΦ cytokine secretion induced by LPS^51^,^52^,^53^,^54^. More generally, glycolysis is important to support DC activation through various PRR to initiate the synthesis of molecules that are critical for cellular functions. In culture, less than 10% of DC can be infected by influenza virus so that glucose consumption and lactate production does not vary at the population level (data not shown). However, DC can still detect the virus through various PRR and react by secreting cytokines. Glycolysis inhibition of DCs by 2-DG reduced cytokine secretion triggered by influenza virus detection (Supplementary Table 1). Glycolysis was also found to be essential for anti-viral response triggered by human plasmacytoid DCs^55^. These observations raised the question of whether pathogens that interfere with TLR4 activation could perturb the regulation of glucose metabolism of innate immune cells. Although there is accumulating evidence indicating that cell metabolic profile is crucial for immune cell function, the molecular mechanisms involved in this functional fine-tuning have not been described so far for human monocyte-derived DCs. In murine DCs derived *in vitro* from bone marrow, two different molecular pathways have been depicted for glycolytic reprogramming. TLR4 signalling involving the activation of TBK1-IKKε and AKT kinases was found to control the early increase in glycolysis^27^. The late increase in glycolytic metabolism upon TLR4 stimulation was found to be a survival response of murine inflammatory DCs to maintain their ATP production despite the inhibition of oxidative phosphorylation by NO. production^28^. Although we cannot exclude a contribution of NO. in the metabolic regulation of DCs by TLR4, we were unable to measure significant NO. production by monocyte-derived DCs (data not shown) and this is in agreement with previous results obtained for human MΦs^56^.

Recent studies suggest that influenza-induced lethality and ALI are due to the TLR4-stimulating effects of DAMPs such as OxPAPC and HMGB1, which are both TLR4 agonists. *In vitro*, HMGB1 can stimulate monocyte-derived DCs, inducing proinflammatory cytokine secretion and FP7 prevented DC activation by HMGB1 (Supplementary Fig. S4). *In vivo*, FP7 not only strongly reduced influenza infection-induced lethality, but also significantly improved the histopathology scores of infected lungs. FP7 prevented excessive inflammation and lung tissue damage and improved the survival rate in this model. FP7 administration also strongly reduced cytokine mRNA and protein production and HMGB1 levels (Table 1) induced by influenza infection. In this model, influenza infection induces a cytokine storm and the accumulation of oxidized products of phospholipids in infected lungs or circulating HMGB1, both of which are inhibited by Eritoran therapy^38^,^39^. Given the inhibitory effect of FP7 on the level of cytokines and HMGB1 induced by PR8 infection, it can be reasonably assumed that the benefit of FP7 treatment of influenza virus–infected mice is linked to its ability to inhibit TLR4 signalling by DAMPs like HMGB1 and the subsequent TLR4-dependent cytokine storm.

The mechanism of action of FP7 has been studied by dissecting its physical interactions with CD14 and TLR4.MD-2^40^. FP7 was designed as a mimetic of lipid X, a biosynthetic precursor of lipid A, and docks well into MD-2 binding cavity^40^. This molecule revealed an unexpectedly high affinity for CD14 and it induces in bone marrow-derived MΦs selective endocytosis of CD14, but not of the TLR4.MD-2 complex^40^. That could impact TLR4 signalling in monocytes and MΦs which express membrane-bound CD14. It was previously shown in murine cells that CD14 promoted LPS-induced TLR4 endocytosis, favouring TRIF-dependent signalling that required internalization of TLR4^5^. These conditions are expected to antagonize TLR4 signalling more effectively than simply competing with LPS for CD14, and TLR4.MD2 binding. Monocyte-derived human DCs do not express membrane-bound CD14 and although soluble CD14 present in the serum can serve as a co-factor for LPS binding on TLR4, enhancing both MyD88- and TRIF-dependent signalling^57^, this soluble receptor cannot trigger internalization. This difference with monocytes may explain why the IC_50_ of FP7 is slightly higher for DCs than for monocytes. Synthesis of FP7 which is a diacylated monosaccharide requires a short procedure giving high yield that is suitable for scale-up. The good bioavailability, high water solubility, lack of toxicity, and selective TLR4 activity make FP7 an ideal compound to be developed as a new drug that targets TLR4-related microbial and sterile inflammatory diseases.

## Methods

### Reagents

Unless otherwise indicated, all chemicals were from Sigma-Aldrich (Saint-Quentin Fallavier, France), and cell culture reagents were from Gibco (ThermoFisher Scientific, Saint-Aubin, France).Ultrapure TLR4 ligand LPS from *E. coli O111:B4*, TLR2/6 ligand peptidoglycan from *S. aureus* (PGN), synthetic TLR1/2 ligand Pam_3_CSK_4_ (Pam3) and poly(I:C) (pIC) were all from InvivoGen (Toulouse, France) and dissolved in sterile PBS. Neutralizing mouse IgG monoclonal anti-human TLR4 and non-blocking control antibodies were kindly provided by Dr. F. Neumann, Innaxon Biosciences. Neutralizing mouse IgA_2_ anti-human TLR4 and non-specific isotype control antibodies were purchased from InvivoGen. FP7 was synthesized from commercially available D-glucose by multistep organic synthesis according to published procedures^40^. The purity of the molecule was assessed by NMR and mass spectrometry analysis^40^. A 5 mM stock solution of FP7 was prepared in ethanol/DMSO 1:1 and was kept at −20 °C for *in vitro* studies. Stock for *in vivo* treatment of mice was prepared in endotoxin-free water (7.5 mg/ml) and then diluted in saline for injection. Eritoran was kindly provided by Eisai Inc. and was prepared as described previously^38^.

### Monocyte purification and treatment

Monocytes were purified as previously described^44^ from human peripheral blood of healthy donors, obtained from the Etablissement Français du Sang with the written informed consent of each donor. All experiments were performed in accordance with the ethical guidelines of the Declaration of Helsinki. Experimental procedures were approved by the local institutional review board (“Comité de Protection des Personnes” Auvergne-Rhône-Alpes). Briefly, peripheral blood mononuclear cells were isolated by standard density gradient centrifugation on Ficoll-Hypaque (Eurobio, Courtaboeuf, France). Mononuclear cells were separated from peripheral blood lymphocytes (PBL) by centrifugation on a 50% Percoll solution (GE Healthcare, Villacoublay, France). Monocytes were purified by immunomagnetic depletion using pan-mouse IgG Dynabeads (ThermoFisher Scientific) with a cocktail of monoclonal antibodies anti-CD19 (4G7 hybridoma), anti-CD3 (OKT3 hybridoma, ATCC, Manassas, VA, USA) and anti-CD56 (NKH1, Beckman Coulter, Fullerton, CA, USA). Monocyte purity was >90% as assessed by CD14 labelling without CD3^+^, CD19^+^ and CD56^+^ contaminating cells (data not shown).

Monocytes were resuspended at 1 × 10^6^ cells/ml in RPMI 1640 medium with GlutaMAX supplemented with 10% foetal calf serum (FCS, BioWhittaker, Lonza, Belgium) and 40 μg/ml gentamycin. They were immediately treated at 37 °C under 5% CO_2_ atmosphere, with FP7 or its solvent for 15 min before addition of LPS or the same volume of PBS as a control. Cells and supernatants were collected after 24 h.

### DC generation and treatment

Monocytes were differentiated to immature DCs during 6 days with human recombinant GM-CSF and IL-4 (Human DC cytokine package, Peprotech, Neuilly-Sur-Seine, France) in RPMI 1640 medium with GlutaMAX, supplemented with 10% FCS and 40 ng/ml gentamycin (complete medium). DCs were harvested, washed twice, and resuspended at 1 × 10^6^ cells/ml of fresh complete medium. DCs were >95% pure as assessed by CD14 and CD1a labelling. Cells were treated at 37 °C under 5% CO_2_ atmosphere with FP7 or its solvent for 15 min before addition of LPS or the same volume of PBS as control for 24 h. Where mentioned, DCs were pre-treated at 37 °C with neutralizing anti-TLR4 or isotype control antibody for 1 h or with 2.5 mM 2-Deoxy-D-Glucose (2-DG) for 15 min before LPS. All cells and supernatants were collected at day 7.

### HEK293/hTLR cell lines

HEK293 cells stably transfected with a plasmid that constitutively express human TLR-related genes were from Invivogen and maintained under antibiotic selection. HEK293/hTLR4-MD2-CD14, HEK293/hTLR1-TLR2 and HEK293/hTLR2-TLR6 were seeded in 96-well plates in 200 μl DMEM medium with L-glutamine, supplemented with 10% FCS and 1% Penicillin/Streptomycin. The following day, cells were treated or not with selected TLR ligands for 24 h. Supernatants were collected and centrifuged before storage.

### Flow cytometry

Monocyte and DC phenotypes were analysed on a FACSCanto II (BD Biosciences, Le Pont de Claix, France) using FITC-conjugated anti-CD14, -CD19, -HLA-DR, -CD80, -CD54, and PE-conjugated anti-CD3, -CD56, -CD1a, -CD86, -CD83 and -CD40 (Beckman Coulter). Viability of cells was monitored using propidium iodide staining of dead cells.

### Cytokine assays

Clarified culture supernatants were stored at −20 °C. IL-8, MIP-1β, IL-6, IL-12 p40, IL-10, and TNF-α in monocyte or DC supernatants were assayed using quantitative cytokine specific Cytometric Bead Array (CBA) Flex Sets (BD Biosciences).

### Glucose consumption and lactate production

Metabolites were quantified in cell supernatants and control medium using glucose (HK) and lactate assay kits (Sigma-Aldrich) according to the manufacturers’ instructions and quantifications were normalized to cell count.

### Mice

Six to 8-week old, WT C57BL/6 J mice were purchased from the Jackson Laboratory (Bar Harbor, ME, USA). All mice were housed and bred in specific pathogen-free conditions. Experiments were conducted in accordance with the guidelines set forth by the University of Maryland, School of Medicine and approved by the institute’s Institutional Animal Care and Use Committees (IACUC).

### Virus

For *in vivo* experiments, mouse-adapted H1N1 influenza A/PR/8/34 virus (“PR8”) (ATCC, Manassas, VA, USA) was grown in the allantoic fluid of 10-day old embryonated chicken eggs as described^58^ and was kindly provided by Dr. Donna Farber (Columbia University).

For *in vitro* experiments, the epidemic influenza A/H1N1/PR/8/34 strain was propagated in Madin-Darby canine kidney (MDCK) cells in DMEM supplemented with 1 μg/ml modified trypsin TPCK (Sigma) in absence of FCS. Virus stocks were titrated by standard plaque assay on MDCK cells using an agar overlay medium.

### Virus challenge and treatment

C57BL/6 J WT 6–8 week old mice (5 mice/treatment group) were infected with mouse-adapted influenza virus, strain A/PR/8/34 (PR8; ~7500 TCID_50_, i.n., 25 μl/nares). This dose was found to kill ~90% of infected WT mice, with no differences in susceptibility between male and female mice (data not shown). Two days after infection, mice received either vehicle or FP7 (200 μg/mouse in 100 μl, i.v.) daily (Day 2 to Day 6). Mice were monitored daily for survival for 14 days. In some experiments, mice were euthanized at day 7 post-infection for analysis of gene expression or histopathology of lungs or for measurement of lung cytokine levels or virus titers in lung homogenates. The number of mice per treatment per experiment was based on a power analysis, with each experiment repeated at least once.

### Histopathology Method

Lungs were inflated and perfused and fixed with 4% paraformaldehyde. Fixed sections (8 mm) of paraffin-embedded lungs were stained with haematoxylin and eosin. Slides were randomized, read blindly, and examined for tissue damage, necrosis, apoptosis, and inflammatory cellular infiltration.

### Quantitative real-time PCR (qRT-PCR)

Total RNA isolation and qRT-PCR were performed on lungs of infected mice as previously described^38^,^59^. Levels of mRNA for specific genes are reported as relative gene expression normalized to mock-infected lungs.

### Cytokine and HMGB1 measurements in lung homogenates

Cytokine protein levels were measured from lung homogenates of PR8-infected mice that were treated either with vehicle or with FP7 and harvested on day 7 post-infection. The right inferior lobe was extracted and homogenized in 1 ml of viral buffer (HBSS with 1% penicillin/streptomycin, 1% Amphotericin B and 10% Sucrose Phosphate Glutamate). Murine IL-1β, TNF-α, IL-6, and KC cytokine protein levels were quantified by Luminex (University of Maryland, Cytokine Core Laboratory). IFN-β was measured using a custom ELISA as previously described^60^. HMGB1 levels were measured by ELISA (IBL International, Hamburg, Germany).

### Virus titration

Virus titers were obtained from supernatants of lung homogenates of PR8-infected mice that were treated either with vehicle or with FP7 and harvested on day 7 post-infection and expressed as TCID_50_/ml as described previously^38^.

### Statistical analysis

Data are expressed as mean ± SEM. The Student’s *t* test was used for comparisons of two sample means. Multiple group comparisons were performed by one-way or two-way ANOVA followed by Bonferroni multiple comparisons test and was applied to the analysis of all dose-dependent experiments. A p-value less than 0.05 was considered statistically significant.

## Additional Information

**How to cite this article**: Perrin-Cocon, L. *et al*. TLR4 antagonist FP7 inhibits LPS-induced cytokine production and glycolytic reprogramming in dendritic cells, and protects mice from lethal influenza infection. *Sci. Rep.* **7**, 40791; doi: 10.1038/srep40791 (2017).

**Publisher's note:** Springer Nature remains neutral with regard to jurisdictional claims in published maps and institutional affiliations.

## Supplementary Material

Supplementary Information

## Figures and Tables

**Figure 1:**
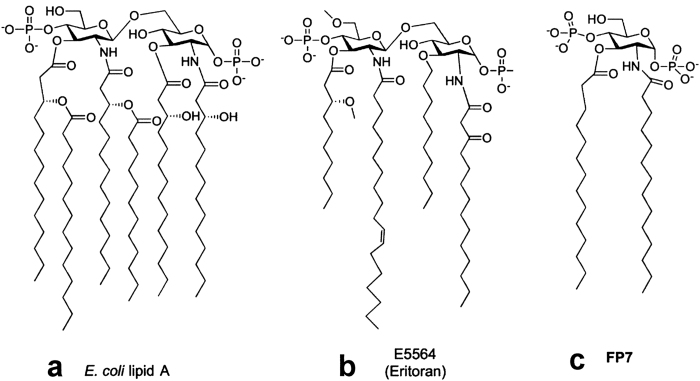
Chemical structures of *E. coli* lipid A (**a**), the natural TLR agonist, and of antagonists Eritoran (**b**; E5564) and monosaccharide FP7 (**c**).

**Table 1:** Cytokine protein analysis of influenza-infected mouse lungs^a^.

Treatment Group	IL-1β (pg/ml)	TNF-α (pg/ml)	IFN-β (pg/ml)	IL-6 (pg/ml)	KC (pg/ml)	HMGB1^e^ (ng/ml)
Mock^b^	17.3 ± 2.6	12.2 ± 1.7	<8^d^	7.4 ± 1	11.1 ± 0.6	—
Vehicle^c^	163.3 ± 23	94.1 ± 5	68.9 ± 8.7	57.5 ± 5.6	119.5 ± 10	5865 ± 169
FP7^c^	50.1 ± 4.9	39.3 ± 7	<8^d^	29.3 ± 4.6	36.7 ± 4.3	4271 ± 504^*^

^a^Mice were treated as described in the legend to Fig. 7, with the addition of a “mock”-infected, untreated group of mice that were not treated (n = 2). On day 7 post-infection, lung homogenates were prepared and cytokines measured by Luminex, except for IFN-β that was measured by ELISA.

^b^Results represent mean ± S.D. for the indicated proteins (n = 2 mice).

^c^Results represent mean ± S.E.M. for the indicated proteins (n = 5 mice/treatment group).

^d^Limit of detection for assay.

^e^HMGB1 was detected in these same lung homogenates by ELISA. *p < 0.007 (Student’s *t* test).
