# Supplementary material for: TLR4 antagonist FP7 inhibits LPS-induced cytokine production and glycolytic reprogramming in dendritic cells, and protects mice from lethal influenza infection
Source: Sci Rep. 2017 Jan 20;7:40791. doi: 10.1038/srep40791 (PMC5247753; doi:10.1038/srep40791)
Supplement: Supplementary Information [file srep40791-s1.pdf]

TLR4 antagonist FP7 inhibits LPS-induced cytokine production and glycolytic reprogramming in dendritic cells, and protects mice from lethal influenza infection

Laure Perrin-Cocon[1,2,3,4,5,*], Anne Aublin-Gex [1,2,3,4,5], Stefania E. Sestito[6], Kari Ann Shirey[7], Mira C. Patel[8], Patrice André [1,2,3,4,5], Jorge C. Blanco[8], Stefanie N. Vogel[7], Francesco Peri[6,*] and Vincent Lotteau[1,2,3,4,5,*]

Supplementary methods

Influenza virus infection of human cells

A549 cells were washed twice with Dulbecco's modified Eagle's medium (DMEM) and infected with the human influenza A/H1N1/PR/8/34 strain (H1N1/PR8) at indicated MOI in infection medium (DMEM supplemented with 0.2 µg/ml TPCK-trypsin (Sigma)). After 1 h at 37°C, the inoculum was discarded and cells were washed again and incubated in fresh medium at 37°C under 5% CO_2.

Monocyte-derived DCs were harvested, numerated, seeded in 24-well plates at 10^6 cells/ml in DC medium (RPMI/ 10% FCS/ 40 µg/ml gentamycin) and incubated with the human H1N1/PR8 influenza virus at indicated MOI. After 24 h incubation at 37°C under 5% CO_2, cells and supernatants were harvested.

Effect of HMGB1

Disulfide, LPS-free HMGB1 was purchased from IBL International (Hamburg, Germany). Monocyte-derived DCs were treated with 2 µg/ml HMGB1 for 24 h in the presence or not of 10 µM FP7.

Supplementary Figures and Table

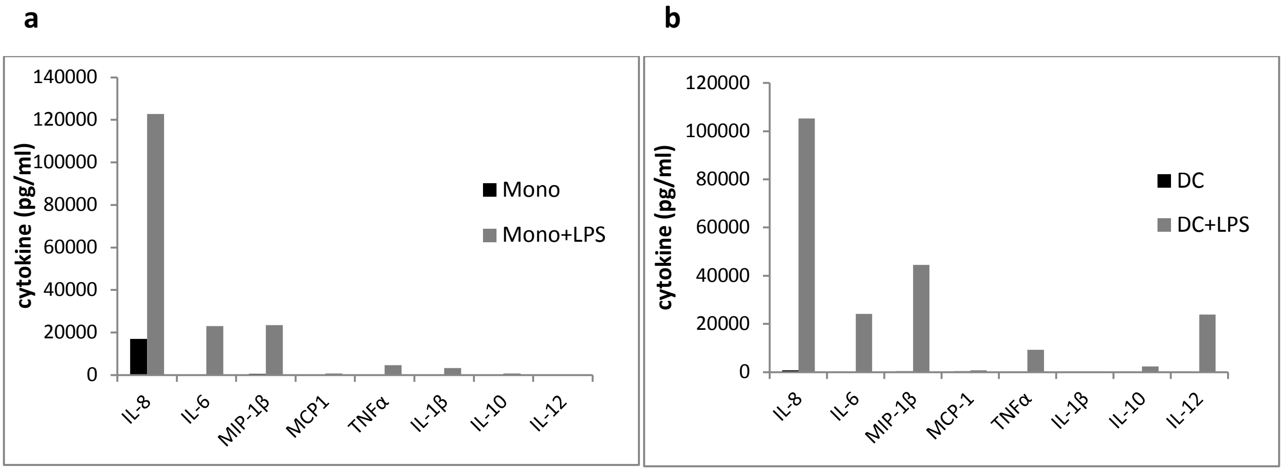

Supplementary Fig. S1. Cytokine secretion panel by monocytes or DCs. Human monocytes freshly isolated from peripheral blood were seeded at 1 x 10^6 cells/ml in fresh medium and stimulated or not for 24 h with 1 µg/ml LPS (a). DCs differentiated for 6 days from monocytes were washed, seeded at 1 x 10^6 cells/ml in fresh medium and stimulated or not for 24 h with 1 µg/ml LPS (b). Cytokines secretion was assayed in cell supernatants by Cytometric Bead Array (CBA, BD Biosciences).

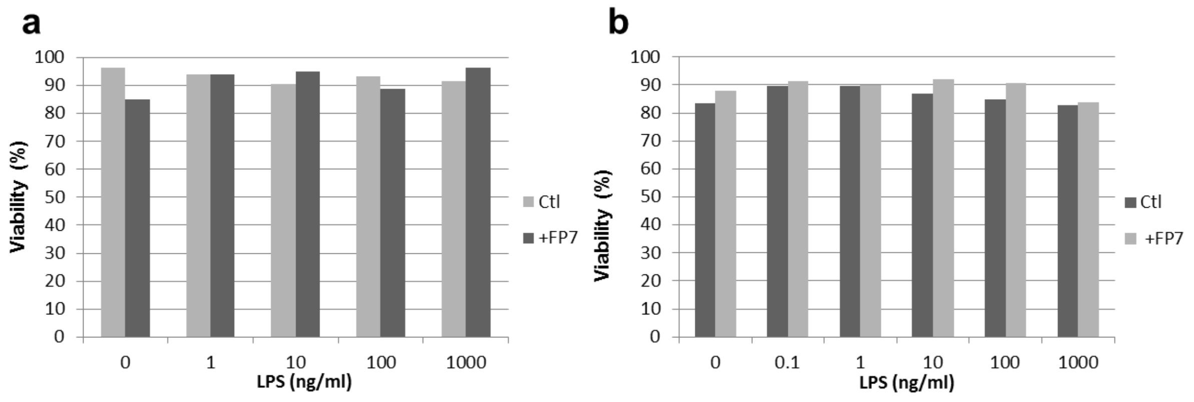

Supplementary Fig. S2. Viability of FP7-treated cells. Human monocytes (a) or DCs (b) were treated with 10 µM FP7 or solvent (Ctl) for 15 min before stimulation for 24h by increasing amounts of LPS. Cells were collected and analysed by flow cytometry. The percentage of viable cells was measured after PI staining of dead cells.

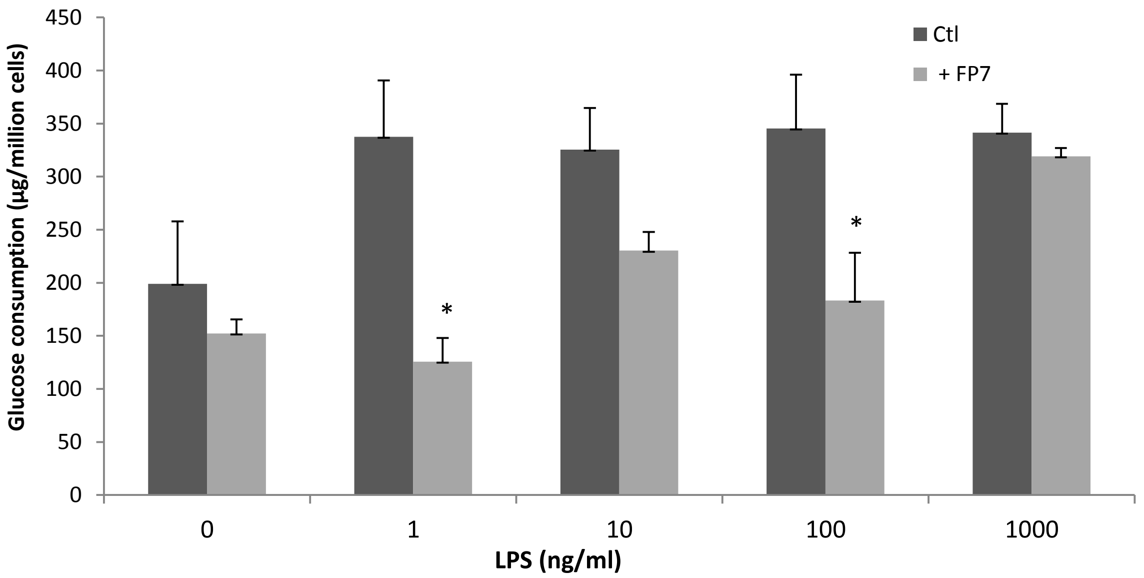

Supplementary Fig. S3. TLR4 modulation of glucose consumption in human monocytes. Human monocytes freshly isolated from peripheral blood were seeded at 1 x 10^6 cells/ml in fresh medium and stimulated for 24 h with increasing amounts of LPS, in the presence of 10 µM FP7 or solvent as control (Ctl). Glucose consumption was monitored using enzymatic detection kits. Results from a representative experiment of 4 are shown.

* $P < 0.05$

	IL-6	IL-8	MIP-1β	IL-10	TNFα	IL-12
**DC ni**	9.2	1349.7	313.7	0.35	0.87	3.81
**DC H1N1/PR8**	1658.2	2205.4	2984.3	1.665	157.23	13.51
**DC 2-DG +H1N1/PR8**	935.8	2011.45	84.05	0.38	88.86	2.705

Supplementary Table 1. Increased secretion of cytokines by DCs incubated with influenza A H1N1/PR8. Human monocyte-derived DCs were seeded at 10^6 cells /ml and incubated in the presence or not of 2.5 mM 2-DG for 24 h with or without (DC ni) H1N1/PR8 viral particles (MOI=1) produced by MDCK cells. Cell supernatants were collected after 24h and cytokines were assayed by CBA. Results expressed in pg/ml are from a representative experiment of two.

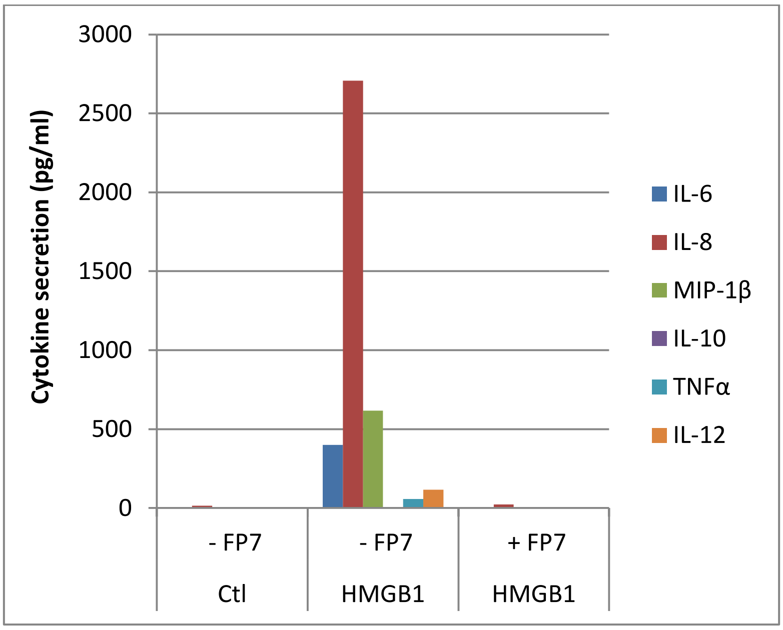

Supplementary Fig. S4. FP7 antagonizes HMGB1 mediated activation of DCs. Monocyte-derived DCs were seeded at 10^6 cells/ml, treated with 10 µM FP7 or control solvent (- FP7), 15 min prior addition of 2 µg/ml disulfide, LPS free HMGB1 or PBS as control (Ctl). After 24 h, cell supernatants were collected and cytokines were assayed by CBA. Results representative from 1 experiment of 2 are shown.
